# Prostate neuroendocrine carcinoma successfully treated with etoposide and cisplatin

**DOI:** 10.1002/iju5.12787

**Published:** 2024-09-22

**Authors:** Masanori Ishizaki, Masaru Hasumi, Kazumichi Muramatsu, Nobuaki Shimizu

**Affiliations:** ^1^ Department of Urology Gunma Prefectural Cancer Center Ota Gunma Japan

**Keywords:** cisplatin, etoposide, metastasis, neuroendocrine carcinoma, prostate

## Abstract

**Introduction:**

The frequency of neuroendocrine carcinoma of the prostate is low, accounting for approximately 1%–5% of all prostate cancers. Herein, we report the case of a patient who was diagnosed with prostate neuroendocrine carcinoma and successfully treated with etoposide and cisplatin.

**Case presentation:**

A 74‐year‐old man presented to the urology department with the chief complaint of urinary retention. After a thorough examination, he was diagnosed with neuroendocrine carcinoma of the prostate and treated with etoposide and cisplatin, called “EP therapy.” The patient remained in remission 1 year later.

**Conclusion:**

A patient with neuroendocrine carcinoma of the prostate was successfully treated with etoposide and cisplatin, and the patient remained in remission. Prostate neuroendocrine carcinomas are rare and have poor prognoses. Therefore, information regarding treatments that can improve prognosis is needed.

**Keynote message**

Prostate neuroendocrine carcinoma is a disease with a poor prognosis. Here, we report a rare case in which etoposide and cisplatin therapy was such a success that the initial carcinoma findings nearly disappeared on later imaging. Our report presents the potential of etoposide and cisplatin for treating prostate neuroendocrine carcinoma.

**Abbreviations & Acronyms**
EP: etoposide and cisplatin
MRI: magnetic resonance imaging
NSE: neuron‐specific enolase
proGRP: progastrin‐releasing peptide
PSA: prostate‐specific antigen

## Introduction

Prostate neuroendocrine carcinoma accounts for approximately 1% of all initial prostate cancer diagnoses.^1^ However, the frequency of neuroendocrine differentiation from adenocarcinoma during the course of treatment is reportedly 10%–100%.^1^

We report the case of a patient who was diagnosed with neuroendocrine carcinoma of the prostate and successfully treated with EP.

## Case presentation

A 74‐year‐old man visited his previous physician with the chief complaint of urinary retention. He had no other complaints or relevant medical or family history. MRI revealed a large mass (approximately 164.32 mm × 97.01 mm) that appeared to be primary prostate cancer, with pancreatic metastasis, multiple enlarged lymph nodes, and invasion into the rectum, left ureter, and iliopsoas muscles. The PSA level was 19.57 ng/mL (reference: 0.000–4.000), NSE level was 98.2 ng/mL (reference: <16.3), and proGRP level was 10 100 pg/mL (reference: <81.0). The patient underwent a prostate needle biopsy and was diagnosed with small‐cell neuroendocrine carcinoma of the prostate. A urinary catheter was inserted, and medical castration was performed by the previous physician at the time of diagnosis.

**Fig. 1:**
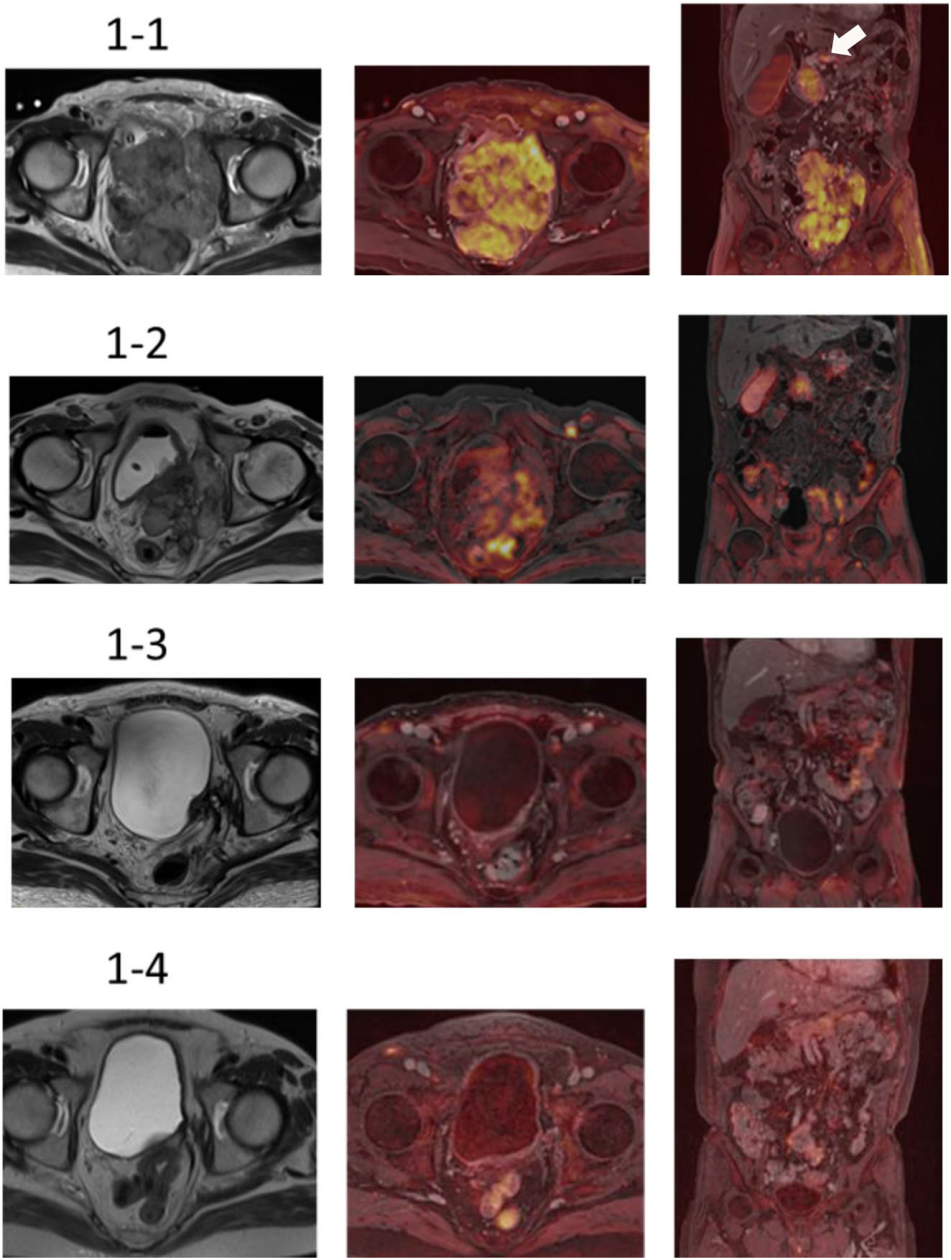
(1‐1) Initial MRI reveals an irregular mass occupying the pelvis, which appears to be primary prostate cancer. The image shows initial evidence of invasion into the surrounding tissues and multiple distant metastases. Arrows indicate pancreatic metastases. (1‐2) MRI after one course of treatment reveals a prominent reduction in tumor size and a trend toward improvement in invasion into the surrounding tissues. (1‐3) MRI after five courses reveals that most of the initial imaging findings have disappeared, including the primary tumor and distant metastases. (1‐4) MRI 1 year after the initial diagnosis. The initial imaging findings are absent.

The patient was referred to our hospital for further evaluation. Blood test results revealed a PSA level of 1.358 ng/mL, NSE level of 146 ng/mL, proGRP level of 14 702 pg/mL, creatinine level of 1.00 mg/dL (reference: 0.65–1.07), and estimated glomerular filtration rate of 56.6 mL/min. No other notable laboratory findings were observed. Computed tomography revealed direct invasion into the bladder, sigmoid colon, iliopsoas muscle, and pelvic wall by prostate cancer and multiple pancreatic and lymph node metastases. MRI revealed an irregular mass occupying the pelvic region, consistent with the findings of perineural invasion and distant metastasis on computed tomography (Fig. 1‐1).

**Fig. 2:**
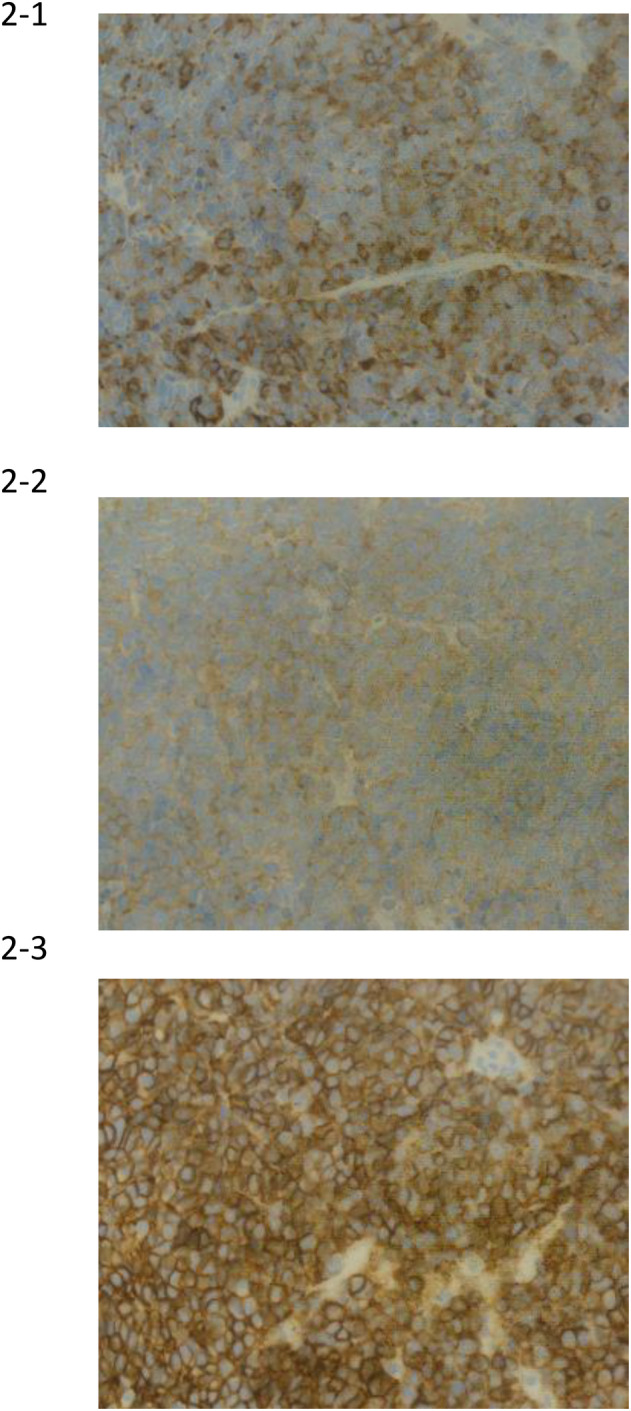
Pathological findings are positive for chromogranin A, synaptophysin, and CD56, which are tumor markers of neuroendocrine carcinoma. (2‐1) chromogranin A. (2‐2) synaptophysin. (2‐3) CD56.

Pathological findings were positive for chromogranin A, synaptophysin, and CD56, which are all tumor markers of neuroendocrine carcinoma (Fig. 2), leading to the diagnosis of small cell neuroendocrine carcinoma. Based on the pathology results, EP therapy was initiated at our hospital. Although medical castration was not performed at our hospital, imaging evaluation after one course of EP showed a reduction in the lesion (Fig. 1‐2), and tumor marker levels declined thereafter. Therefore, five courses of EP were administered within 4 months. Specifically, etoposide was administered at 100 mg/m^2^ for 3 consecutive days, and cisplatin was administered at 80 mg/m^2^ on the first day and repeated every 3 weeks.

**Fig. 3:**
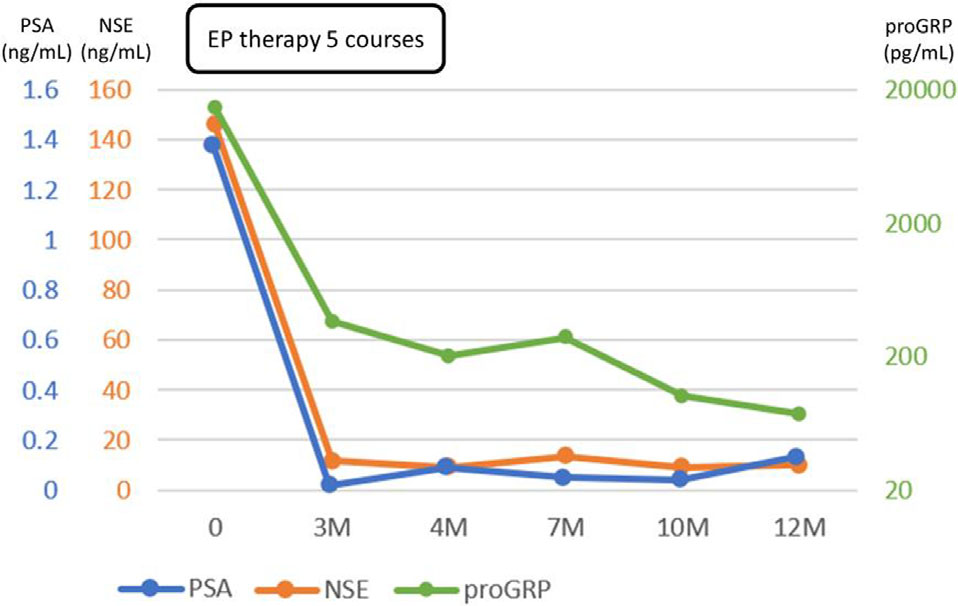
Clinical course and changes in PSA, NSE, and proGRP during the first year of treatment.

MRI after five courses of EP therapy revealed that the primary pelvic mass had markedly shrunk. Additionally, the pancreatic and lymph node metastases had nearly disappeared (Fig. 1‐3). Thereafter, urinary retention improved. The tumor marker levels, with a PSA level of 0.071 ng/mL, NSE level of 9.0 ng/mL, and proGRP level of 202.4 pg/mL, were markedly reduced compared with their values before treatment.

One year after the initial visit, the patient still had good urinary drainage and remained in remission, based on imaging findings (Fig. 1‐4). The levels of NSE and proGRP were 9.9 ng/mL and 73.8 pg/mL, respectively, and the tumor marker levels remained low (Fig. 3).

## Discussion

The frequency of neuroendocrine carcinomas of the prostate is low, accounting for approximately 1%–5% of all prostate cancers.^2^ Small‐cell carcinoma, which was the diagnosis in the present case, is the most common.^3^

The frequency of differentiation from adenocarcinoma to neuroendocrine carcinoma during the course of treatment is reportedly 10%–100%.^1^ However, the frequency of neuroendocrine carcinoma at the time of initial diagnosis, as in the present case, is approximately 1%.^1^

Prostate neuroendocrine carcinoma is rarely detected as a localized cancer; more than 70%–75% of prostate neuroendocrine carcinomas have distant metastases at the time of detection.^4^, ^5^ Therefore, systemic chemotherapy is the mainstay of treatment, although no effective treatment has been established. Chemotherapy similar to that used for small cell lung cancer, such as the EP therapy used in the present case or irinotecan and cisplatin therapy, is often used.^6^ Our patient showed invasion into the surrounding tissues and multiple metastases after the initial diagnosis and was diagnosed with stage IV cT4N1M1c. The patient exhibited prominent rectal involvement. A stoma was considered at the pretreatment stage but was avoided owing to successful treatment.

Some studies have reported successful multidisciplinary treatments, including radiotherapy, for localized cancer.^7^ However, the prognosis for most prostate neuroendocrine carcinomas remains poor. The average survival time is 9.8 months (7.3 months for patients with metastases at the time of initial diagnosis).^5^ Few patients have remained in remission for >1 year, similar to the present patient.

Table 1 lists the cases of patients who have responded to chemotherapy over the last 10 years in Japan (Table 1). In addition to EP, olaparib in patients with BRCA‐positive and pembrolizumab in patients with microsatellite instability‐high have been reported to be successful in a small number of cases.

**Table 1:** Cases of patients reported to have responded to chemotherapy over the last 10 years in Japan

No. (year)	Author	Chemotherapy
1 (2014)	Kataoka	Cisplatin + Irinotecan
2 (2014)	Ishii	Docetaxel
3 (2016)	Yumiba	Carboplatin + Etoposide
4 (2016)	Fujii	Cisplatin + Irinotecan
5 (2016)	Sasaki	Carboplatin + Etoposide
6 (2018)	Tanaka	Carboplatin + Etoposide
7 (2021)	Shimizu	Cisplatin + Etoposide
8 (2022)	Tsuboya	Amrubicin
9 (2023)	Maruyama	Olaparib
10 (2023)	Funaki	Cisplatin + Etoposide
11 (2023)	Samejima	Pembrolizumab
12 (2023)	Miyazawa	Olaparib

For lung cancer, multimodal treatment, including radiotherapy, is recommended for advanced neuroendocrine cancer, and the same is considered for prostate cancer. However, no clear evidence has been presented to date, and it is necessary to consider treatment on a case‐by‐case basis.^8^ There have been cases of response to chemotherapy alone, as shown in Table 1, including the present case. New treatments for prostate neuroendocrine cancer using immune checkpoint inhibitors are being tested in clinical trials, and we look forward to the accumulation of more cases in the future.

## Conclusion

We report a case of neuroendocrine carcinoma of the prostate that responded to EP therapy. The patient was successfully treated with EP and remained in remission 1 year later. Prostate neuroendocrine carcinomas are rare and have poor prognoses. Therefore, information regarding treatments that can improve prognosis is needed.

## Author contributions

Masanori Ishizaki: Conceptualization; data curation; formal analysis; validation; visualization; writing – original draft. Masaru Hasumi: Conceptualization; data curation; formal analysis; investigation; methodology; project administration; writing – review and editing. Kazumichi Muramatsu: Data curation; investigation; methodology; validation. Nobuaki Shimizu: Investigation; methodology; project administration; resources; supervision; writing – review and editing.

## Conflict of interest

The authors declare no conflict of interest.

## Informed consent

Written informed consent was obtained from the patient for publication of this case report.

## Registry and the Registration No. of the study/trial

N/A.

## Approval of the research protocol by an Institutional Review Board

N/A.
